# Parallelizing Backpropagation Neural Network Using MapReduce and Cascading Model

**DOI:** 10.1155/2016/2842780

**Published:** 2016-04-27

**Authors:** Yang Liu, Weizhe Jing, Lixiong Xu

**Affiliations:** School of Electrical Engineering and Information, Sichuan University, Chengdu 610065, China

## Abstract

Artificial Neural Network (ANN) is a widely used algorithm in pattern recognition, classification, and prediction fields. Among a number of neural networks, backpropagation neural network (BPNN) has become the most famous one due to its remarkable function approximation ability. However, a standard BPNN frequently employs a large number of sum and sigmoid calculations, which may result in low efficiency in dealing with large volume of data. Therefore to parallelize BPNN using distributed computing technologies is an effective way to improve the algorithm performance in terms of efficiency. However, traditional parallelization may lead to accuracy loss. Although several complements have been done, it is still difficult to find out a compromise between efficiency and precision. This paper presents a parallelized BPNN based on MapReduce computing model which supplies advanced features including fault tolerance, data replication, and load balancing. And also to improve the algorithm performance in terms of precision, this paper creates a cascading model based classification approach, which helps to refine the classification results. The experimental results indicate that the presented parallelized BPNN is able to offer high efficiency whilst maintaining excellent precision in enabling large-scale machine learning.

## 1. Introduction

At present, big data analysis has become an important methodology in finding data associations [1], whilst classification is one of the most famous research methods. Among types of classification algorithms, Artificial Neural Network (ANN) is proved to be an effective one that can adapt to various research scenarios. In numbers of ANN implementations, backpropagation neural network (BPNN) is the most widely used one due to its excellent function approximation ability [2]. A typical BPNN usually contains three kinds of layers including input layer, hidden layer, and output layer. Input layer is the entrance of the algorithm. It inputs one instance of the data into the network. The dimension of the instance determines the number of inputs in the input layer. Hidden layer contains one or several layers. It outputs intermediate data to the output layer that generates the final output of the neural network. The number of outputs is determined by the encoding of the classified results. In BPNN each layer consists of a number of neurons. The linear functions or nonlinear functions in each neuron are frequently controlled by two kinds of parameters, weight and bias. In the training phase, BPNN employs feed forward to generate output. And then it calculates the error between the output and the target output. Afterwards, BPNN employs backpropagation to tune weights and biases in neurons based on the calculated error. In the classifying phase, BPNN only executes feed forward to achieve the ultimate classified result. Although it is difficult to determine an optimal number of the hidden layers and neurons for one classification task, it is proved that a three-layer BPNN is enough to fit the mathematical equations which approximate the mapping relationships between the inputs and the outputs.

However, BPNN has encountered a critical issue that, due to a large number of mathematical calculations existing in the algorithm, low efficiency of BPNN leads to performance deterioration in both training phase and classification phase when the data size is large. Therefore to fulfil the potential of BPNN in big data processing, this paper presents a parallel BPNN (CPBPNN) algorithm based on the MapReduce computing model [3] and cascading model. The algorithm firstly creates a number of classifiers. Each classifier is trained by only one class of the training data. However, in order to speed up the training efficiency and maintain generalization, the class of training data does not train only one classifier but a group of classifiers. As long as one testing instance is input into these classifiers, they classify it and output their individual results. Afterwards, a majority voting is executed to decide the final result. If the testing instance is correctly classified, its classification is completed. Otherwise if the testing instance cannot be correctly classified by the classifiers, it will be output to a second group of classifiers trained by another class of training data until all groups of classifiers are traversed. The algorithm is implemented in the MapReduce environment. The detailed algorithm design and implementation are presented in the following sections.

The rest of the paper is organized as follows.  Section 2 presents the related work; Section 3 describes the algorithm design in detail; Section 4 discusses the experimental results; and Section 5 concludes the paper.

## 2. Related Work

It has been widely admitted that ANN has become an effective tool for processing nonlinear function approximation tasks, for example, recognition, classification, and prediction tasks. A number of researches employed neural network to facilitate their researches. Almaadeed et al. introduced a wavelet analysis and neural networks based text-independent multimodal speaker identification system [4]. The wavelet analysis firstly employs wavelet transforms to execute feature extraction. And then the extracted features are used as input for different types of neural networks, which create a number of learning modules including general regressive, probabilistic, and radial basis. Their results indicate that the employed BPNN can classify the data generated by DWT (discrete wavelet transform) and WPT (wavelet packet transform) with high accuracy. Chen and Ye employed a four-layer backpropagation neural network to compute ship resistance [5]. In their research, they studied the impact of algorithm performances with different parameters. Based on their results, with the original ship model experimental data, BPNN can help to develop high-precision neural network systems for the computation of ship resistance. Khoa et al. pointed out that, in the stock price forecasting, it is difficult to generate accurate predictions due to multiple unknown factors [6]. Therefore, they employed a feed forward neural network (FFN) and a recurrent neural network (RNN) to execute the prediction and they also employed the backpropagation mechanism to train and adjust the network parameters.

Recently, neural network with processing large-scale tasks is anxiously needed in big data application. However, the neural networks including BPNN have low efficiency in processing large-scale data. A number of effects have been done by researchers. They mainly focused on tuning the network parameters to achieve high performance. Research [7] combines the neural network algorithm with evolutionary algorithms. The approach can exploit the geometry of the task by mapping its regularities onto the topology of the network, thereby shifting problem difficulty away from dimensionality to the underlying problem structure. Jin and Shu pointed out that BPNN needs a long time to converge [8] so they employed the artificial bee colony algorithm to train the weights of the neural network to avoid the deficiency of BPNN. Li et al. proposed an improved BPNN algorithm with self-adaptive learning rate [9]. The experimental results show the number of iterations is less than that of the standard BPNN with constant learning rate. Also several researchers tried to solve the scale issue with combing cloud computing techniques. For example, Yuan and Yu proposed a privacy preserving BPNN in the cloud computing environment [10]. The authors aimed at enabling multiple parties to jointly conduct the BPNN learning without revealing their private data. The input datasets owned by the parties can be arbitrarily partitioned to achieve a scalable system. Although the researchers claimed that their algorithm supplies satisfied accuracy, they have not conducted the detailed experiments for testing the algorithm efficiency. It is well known that the cloud computing is extremely loosely coupled so that the cloud environment based neural network may encounter a large overhead. Additionally the researches have not mentioned how their algorithm performs in dealing with the practical large-scale tasks. Researches [11–13] stated that a better choice to implement large-scale classification is to parallelize BPNN using the parallel and distributed computing techniques [14]. Research [15] presented three types of Hadoop based distributed BPNN algorithms. A great difference from the work presented by this paper is that, due to the cascading model, our algorithm could improve the algorithm precision. However, the algorithms in [15] can only guarantee but not improve the algorithm precision.

Gu et al. presented a parallel neural network using in-memory data processing techniques to speed up the computation of the neural network. However, their algorithm does not consider the accuracy issue [16]. In this work, the training data is simply segmented into a number of data chunks which are processed in parallel, which may result in accuracy loss. Hebboul et al. also parallelized a distributed neural network algorithm based on the data separation [17]. However, the accuracy loss is also a critical issue in their work. Ganeshamoorthy and Ranasinghe created a vertical partition and hybrid partition scheme [18] for parallelizing neural network using MPI (Message Passing Interface) [19]. However, MPI requires a highly homogeneous environment which decays the adaption of the parallelized algorithm.

The work presented in this paper mainly focuses on parallelizing BPNN in terms of improving the algorithm efficiency, simultaneously maintaining the algorithm classification accuracy in dealing with large-scale data. The paper employs the Hadoop framework as the underlying infrastructure. And then a number of designs have been done in order to improve the algorithm efficiency in both training and classification phases. Also a cascading mechanism is introduced to enhance the algorithm classification accuracy.

## 3. Algorithm Design

### 3.1. Backpropagation Neural Network

**Figure 1:**
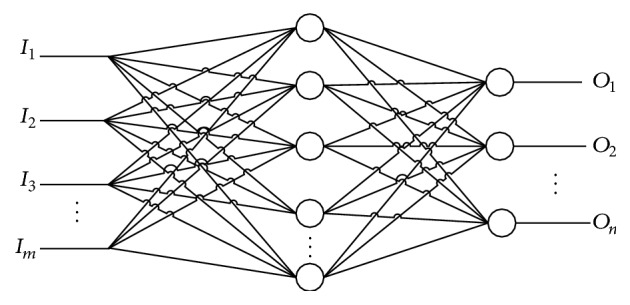
Structure of a three-layer BPNN.

BPNN is a multilayer network including input layer, hidden layer, and output layer. Each layer consists of a number of neurons. In order to adjust the weights and biases in neurons, BPNN employs error backpropagation operation. Benefiting from the gradient-descent feature, the algorithm has become an effective function approximation algorithm [20, 21]. A standard BPNN which consists of a number of *m* inputs and *n* outputs is shown in Figure 1.

In the feed forward, each neuron in the next layer inputs the outputs from all neurons in the last layer. And then it outputs its output which will be input into the next layer neurons. For one neuron *j*, let *n* denote the number of neurons in the last layer; *o*_*i*_ the output of the *i*th neuron; *w*_*i*_ the corresponding weight for *o*_*i*_; *θ*_*j*_ the bias of the neuron *j*. Therefore the neuron *j* calculates the input for the sigmoid function *I*_*j*_ using

(1) $$\begin{array}{c} I_{j}=\underset{n}{\sum }w_{i}o_{i}+\theta_{j}. \end{array}$$

Let *o*_*j*_ denote the output of neuron *j*; it can be represented using

(2) $$\begin{array}{c} o_{j}=\frac{1}{\left(1+e^{-I_{j}}\right)}. \end{array}$$

If the neuron *j* is in the output layer, BPNN starts the backpropagation phase. Let *t*_*j*_ denote the encoded target output. The algorithm computes the output error Err_*j*_ for the neuron *j* in the output layer using

(3) $$\begin{array}{c} {\text{Err}}_{j}=o_{j}\left(\;1-o_{j}\;\right)\left(\;t_{j}-o_{j}\;\right). \end{array}$$

Let *k* denote the number of neurons in the next layer; *w*_*p*_ the weight; and Err_*p*_ the error of neuron *p* in the next layer. The error Err_*j*_ of the *j*th neuron can be represented using

(4) $$\begin{array}{c} {\text{Err}}_{j}=o_{j}\left(\;1-o_{j}\;\right)\underset{k}{\sum }{\text{Err}}_{p}w_{p}. \end{array}$$

Following, let *η* denote the learning rate. The neuron *j* tunes its weight *w*_*j*_ and bias *θ*_*j*_ using

(5) Δwj=ηErrjoj,Δθj=ηErrj,wj=wj+Δwj,θj=θj+Δθj.

When BPNN finishes tuning the network with one training instance, it starts to input a second training instance until all the training instances are processed. In order to execute the classification, BPNN needs to only execute the feed forward. The outputs at the output layer are the final classification result.

### 3.2. MapReduce and Hadoop

**Figure 2:**
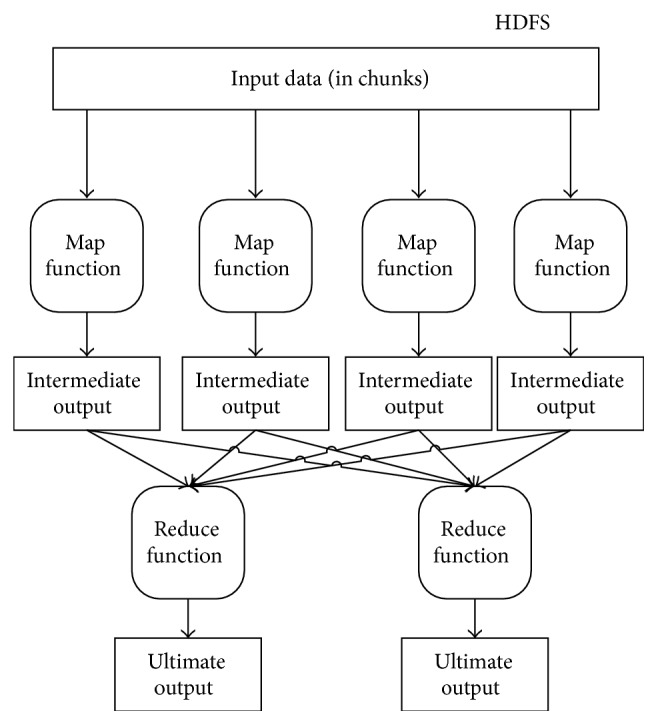
MapReduce model.

MapReduce is a distributed computing model which contains two main operations Map and Reduce. The Map operation inputs each data record in the form of key-value pair, for example, {Key1, Value1}. And then the Map executes computations and outputs the intermediate output in key-value pair {Key2, Value2}. The Reduce operation collects the intermediate outputs from all the Maps. Afterwards it merges and sorts the data records based on the keys and finally it generates the ultimate result [22].  Figure 2 shows how the MapReduce computing model works.

Hadoop framework [25, 26] is a Java based implementation of the MapReduce computing model. In one Hadoop cluster the nodes are categorized into one NameNode and several DataNodes. The NameNode manages the metadata of the cluster, whilst the DataNode executes a number of Map (mapper) and Reduce (reducer) operations in parallel. Both the NameNode and DataNodes contribute their resources including processors, memory, hard disks, and network adaptors to form Hadoop Distributed File System (HDFS) [27]. HDFS is not only responsible for high performance data storage but also managing data processing courses for the mappers and reducers. The resource management in HDFS is controlled by Yarn [25], so that HDFS supplies a number of advanced features including data replication, fault tolerance, load balancing, data compression, and heterogeneous hardware support.

### 3.3. Parallelizing BPNN

The low efficiency issue of BPNN frequently occurs in the training and classification phases. If the volume of training data is large, the overhead of the algorithm deteriorates the performance. On the other hand, if the volume of the to-be-classified data is large, the algorithm may also perform worse. The presented parallelized BPNN (CPBPNN) considers the efficiency improvement for both the training and classification phases.

#### 3.3.1. Parallelization in Training

This section mainly focuses on speeding up the training phase in BPNN. The parallelization for the training phase is based on the data separation. Let *T* denote the training data; *a* the number of BPNNs (sub-BPNNs); *T*_*i*_ the *i*th divided data chunk. To parallelize the training, *T* can be separated into a number of *a* chunks:

(6) $$\begin{array}{c} \overset{}{\underset{a}{⋃}}\;T_{i}=T. \end{array}$$

Each sub-BPNN inputs one chunk and starts training. As a result, each sub-BPNN becomes a trained classifier which can be employed in future classifications. Based on the data separation and the sub-BPNNs, the training phase can be accelerated. However, the simple data separation causes one issue that as each sub-BPNN is only trained by a part of the original training data, the less number of training instances could impact the classification precision. Therefore, our work introduces the ensemble techniques including bootstrapping and majority voting to solve the issue.

Bootstrapping [28] is based on the idea of controlling the number of times that the training instances appear in the bootstrap samples, so that in the *B* bootstrap samples, each instance appears the same number of times. The most efficient way of creating balanced bootstrap samples is to construct a string of the instances *X*_1_, *X*_2_, *X*_3_,…, *X*_*n*_ repeating *B* times so that a sequence of *Y*_1_, *Y*_2_, *Y*_3_,…, *Y*_*Bn*_ is achieved. A random permutation *p* of the integers from 1 to *B*_*n*_ is taken. Therefore the first bootstrapping sample can be created from *Y*_*p*_(1), *Y*_*p*_(2), *Y*_*p*_(3),…, *Y*_*p*_(*n*), moreover the second bootstrapping sample from *Y*_*p*_(*n* + 1), *Y*_*p*_(*n* + 2), *Y*_*p*_(*n* + 3),…, *Y*_*p*_(2*n*), and so on until *Y*_*p*_((*B* − 1)*n* + 1), *Y*_*p*_((*B* − 1)*n* + 2), *Y*_*p*_((*B* − 1)*n* + 3),…, *Y*_*p*_(*Bn*) is the *B*th bootstrapping sample. Assume there are a number of *a* mappers in a Hadoop cluster. Each mapper initializes one sub-BPNN for training. Therefore CPBPNN firstly generates a number of *a* bootstrapped samples. Each sample *T*_*i*_ is saved in a data chunk in HDFS:

(7) bootstrap T⟶T1,T2,T3,…,Ta,⋃i=1a Ti=T.

Let Instance_*b*_ denote one training instance in *T*_*i*_. For facilitating the algorithm design, the Instance_*b*_ is saved in HDFS using a customized data structure:

(8) $$\begin{array}{c} \left\{\;{\text{Instance}}_{b}:\text{target output}:\text{instance type}\;\right\}; \end{array}$$

(i) target output represents the target output, which is the training instance Instance_*b*_ belonged to;
(ii) type is a string marked as “train” or “test,” which explicitly informs CPBPNN with the fact that current instance is a training instance or a to-be-classified instance.

**Figure 3:**
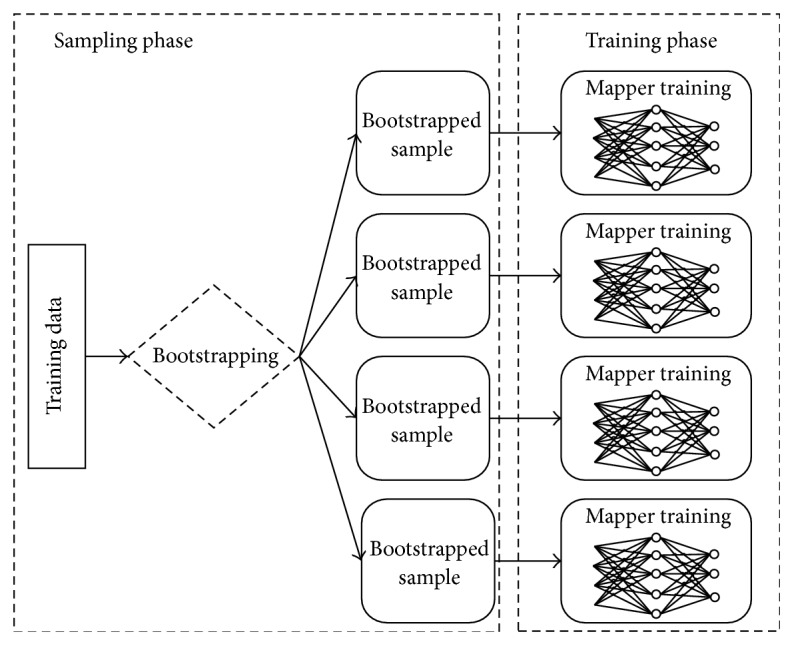
Parallelization in training phase.

When the training phase starts, each mapper creates one sub-BPNN and randomly initializes weight and bias between [−1,1] for every neuron. Afterwards the *i*th BPNN inputs the instances of the data chunk *T*_*i*_. As long as one instance is input, its type is parsed. If the instance type is “train”, the BPNN starts the feed forward and the backpropagation processes using (1) to (5) to tune the network parameters. After all sub-BPNNs in mappers finish their training, a group of weak classifiers are created.  Figure 3 shows the parallelization for one group of weak classifiers.

*Parallelization in Training*

(1) Each mapper constructs one BPNN with 3 layers.
(2) Initialize *w*, *θ* ∈ [−1,1] for each neuron randomly.
(3) Bootstrap {*T*_1_, *T*_2_, *T*_3_,…, *T*_*a*_},  ⋃_*i*=1_^*a*^ *T*_*i*_ = *T*, *T*_*i*_ for mapper_*i*_.
(4) Each mapper inputs one training instance in *T*_*i*_ and computes
  (9) Ij=∑nwioi+θjoj=11+e−Ij.
(5) In output layer, backpropagation computes
  (10) $$\begin{array}{c} {\text{Err}}_{j}=o_{j}\left(\;1-o_{j}\;\right)\left(\;t_{j}-o_{j}\;\right). \end{array}$$
(6) In other layers, backpropagation computes
  (11) $$\begin{array}{c} {\text{Err}}_{j}=o_{j}\left(\;1-o_{j}\;\right)\underset{k}{\sum }{\text{Err}}_{p}w_{p}. \end{array}$$
(7) Update *w*, *θ* using (5).

Repeat (4), (5), (6), and (7).

Until instances in *T*_*i*_ are processed, training terminates.

#### 3.3.2. Parallelization in Classification

In the scenario of processing the large volume of classification data, BPNN also encounters the low efficiency issue as BPNN classifies the instances one by one, which generates large IO and calculation overheads. CPBPNN also considers the parallelization for the to-be-classified data. The parallelization is based on the data separation. Let *g* denote the number of weak classifier groups and *C* the data to be classified. Therefore, *C* can be separated into *g* chunks:

(12) $$\begin{array}{c} \overset{}{\underset{g}{⋃}}{\;C}_{i}=C. \end{array}$$

Each chunk *C*_*i*_ is input into a weak classifier group *i* ∈ *g*. In the *i*th group, majority voting is employed to classify the instances. Majority voting [28] is a commonly used combination technique. The ensemble classifier predicts a class for an instance using the majority of base classifiers [29, 30]. The classification phase employs only feed forward based on (1) and (2) to do the classification. If the type of one instance in *C*_*i*_, for example, the Instance_*c*_ is labeled as “test”, it will be input into each mapper. The sub-BPNNs in the mappers generate their own classified results of the Instance_*c*_ at the output layer. And then the mappers output intermediate outputs in the form of

(13) $$\begin{array}{c} \left\{\;{\text{Instance}}_{c},o_{m}\;\right\}, \end{array}$$

where Instance_*c*_ is the key and  *o*_*m*_ is the output of the *m*th mapper.

When all the mappers finished their outputs, one reducer starts collecting the outputs of the mappers. The outputs with the same key are merged together, which forms a set similar to (assuming 6 outputs from mappers with 3 values)

(14) $$\begin{array}{c} \left\{\;{\text{Instance}}_{c},o_{1}\left(\;{\text{value}}_{1}\;\right)\;\right\},\\ \left\{\;{\text{Instance}}_{c},o_{2}\left(\;{\text{value}}_{1}\;\right)\;\right\},\\ \left\{\;{\text{Instance}}_{c},o_{5}\left(\;{\text{value}}_{1}\;\right)\;\right\},\\ \left\{\;{\text{Instance}}_{c},o_{6}\left(\;{\text{value}}_{1}\;\right)\;\right\},\\ \left\{\;{\text{Instance}}_{c},o_{3}\left(\;{\text{value}}_{2}\;\right)\;\right\},\\ \left\{\;{\text{Instance}}_{c},o_{4}\left(\;{\text{value}}_{3}\;\right)\;\right\}. \end{array}$$

**Figure 4:**
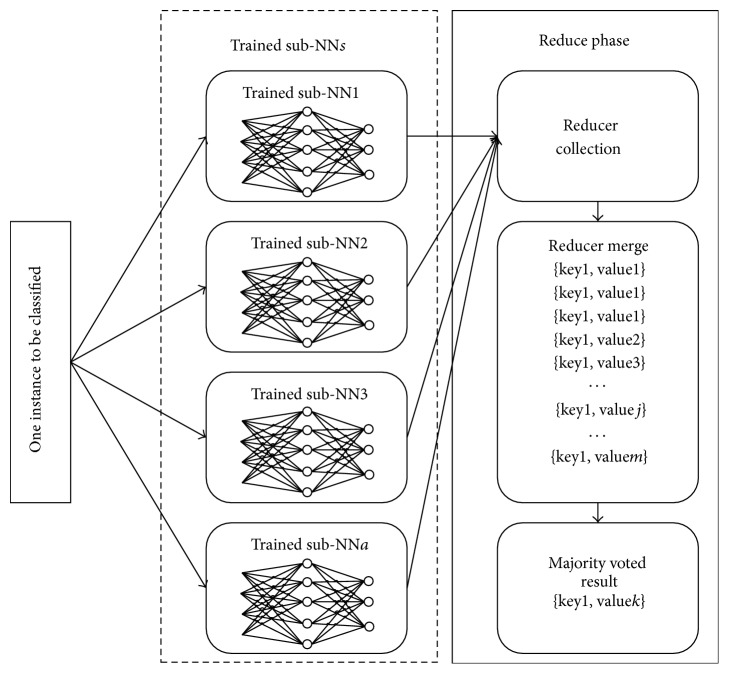
Classifying one instance in classification phase.

The reducer runs the majority voting and outputs the final result for the Instance_*c*_ into HDFS in the form of {Instance_*c*_, *r*_*c*_} where *r*_*c*_ represents the voted final classification result of the Instance_*c*_. Based on the bootstrapping and majority voting, a number of weak classifiers can form a strong classifier, which improves the training efficiency whilst guaranteeing the algorithm accuracy.  Figure 4 shows the process of classifying one instance in the classification phase.

*Classifying One Instance in Classification Phase*

(1) Each mapper inputs testing instance Instance_*c*_ ∈ *C*_*i*_.
(2) BPNN in each mapper executes feed forward:
  (15) Ij=∑nwioi+θjoj=11+e−Ij.
(3) Mapper outputs {Instance_*c*_, *o*_*m*_},  *m* = {1,2, 3,…, *a*}.
 Reducer collects {Instance_*c*_, *o*_*m*_}.
 Reducer executes majority voting and output {Instance_*c*_, *r*_*c*_}.

Classification terminates.

#### 3.3.3. Classification Accuracy Improvement

CPBPNN employ a cascading model to improve the classification precision. The design of the algorithm is presented as follows:

(1) Let cn represent the number of classes in the training data; class_*i*_ represents the *i*th class. CPBPNN employs a number of *a* mappers to initiate the number of *a* sub-BPNNs. And then the number of *a* mappers is grouped into a number of *g* groups. The sub-BPNNs in each group are trained using class_1_.
(2) CPBPNN separates the to-be-classified data into the number of *g* chunks. In each group generated in step (1), one classifier sc_*i*_ classifies the instances of the chunk* C*_*i*_. As the sc_*i*_ is only trained by the data from class_1_, it can only do the classification for one class. Therefore the successfully classified instances are filtered and output as the result. The criteria for determining if one instance is correctly classified are to compare the classification output to the class of the training instances. If the classification output equals the class of the training instances, the instance is correctly classified. Otherwise, the instance is not correctly classified. The instances that cannot be correctly classified are output as errorset_1_. The error set contains a number of unclassified instances, which will be used as input for the second-round classification.
(3) And then the classifier sc_*i*_ trains its sub-BPNNs using class_2_. Following it inputs and classifies the instances in errorset_1_. In this case, the instances in errorset_1_ that belonged to class_2_ can be recognized. They will be filtered and output as the classification result. The instances that cannot be correctly classified are output as errorset_2_.
(4) Finally the classifier sc_*i*_ trains itself until the class class_cn_ is input. And then it outputs the classified instances. The unsuccessfully classified instances are output as errorset_cn_.
(5) If errorset_cn_ is empty, all instances in the* C*_*i*_ are classified. Otherwise, the instances in errorset_cn_ cannot be classified into any class.

**Figure 5:**
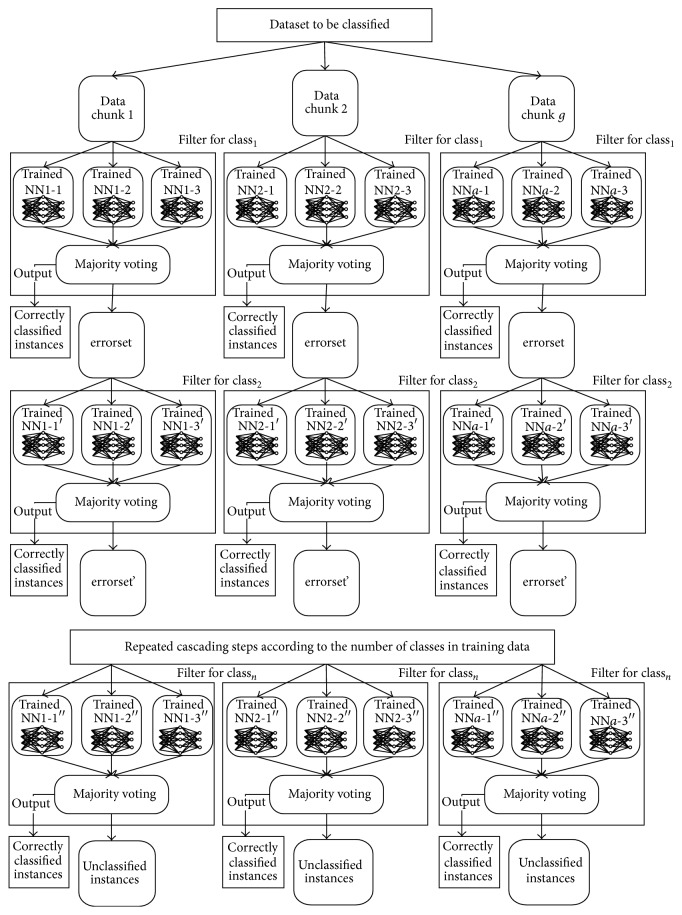
CPBPNN structure.

Figure 5 shows the entire structure of CPBPNN.

## 4. Algorithm Evaluation

In order to evaluate the performance of CPBPNN, a Hadoop cluster is established. The cluster contains five nodes. One is NameNode and the other four nodes are DataNodes. The details of the cluster are listed in Table 1.

The datasets for the performance evaluation mainly contain Iris dataset [31] and Wine dataset [32]. Both datasets are regarded as standard benchmark datasets in machine learning field. The details of the datasets are listed in Table 2.

There are 15 mappers and 3 reducers employed to execute the training and classification tasks. As input layer of each sub-BPNN with 3 layers in one mapper only accepts the value between 0 and 1, therefore the input instances should be normalized in advance. For one instance instance_*k*_ = {*a*_1_, *a*_2_, *a*_3_,…, *a*_*n*_}, let *a*_max⁡_, *a*_min⁡_, and *na*_*i*_ denote the maximum element, minimum element, and normalized *a*_*i*_ respectively; then

(16) $$\begin{array}{c} na_{i}=\frac{a_{i}-a_{\min}}{a_{\max}-a_{\min}}. \end{array}$$

Let rightNum represent the number of correctly classified instances and wrongNum the number of wrongly classified instances. Therefore the classification precision *p* is

(17) $$\begin{array}{c} p=\frac{\text{rightNum}}{\text{rightNum}+\text{wrongNum}}\times 100\%. \end{array}$$

### 4.1. Precision

**Figure 6:**
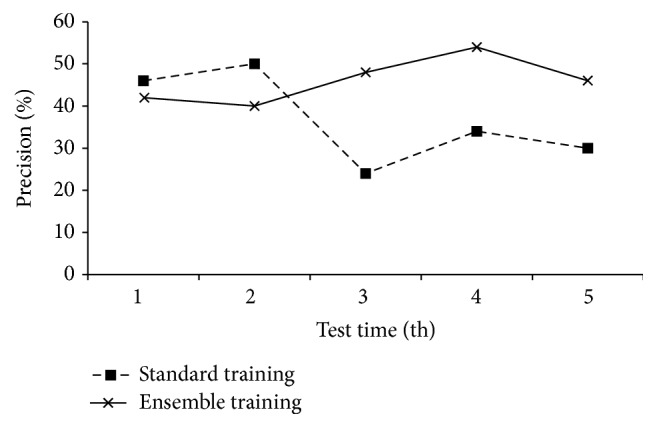
Comparison of standard training in BPNN and ensemble training.

The first evaluation is to observe the performance of the presented training strategy, which trains the neural network in parallel but with less accuracy loss. In the evaluation, a small number of training instances are randomly selected from the Iris dataset. We also randomly selected 50 instances from the Iris dataset as testing data. For observing the comparison in terms of precision, a standard BPNN training and a standalone ensemble training are both implemented.  Figure 6 shows the evaluation result.

Figure 6 indicates that the standard training of BPNN products unstable performances in five times test. The accuracy fluctuates severely. Contrarily, the ensemble based training gives correspondingly stable performances. The mean square deviation of the standard training is 10.92, whilst the one of the ensemble training is only 5.48. And also the average precision of the ensemble training is higher than that of the standard training.

**Figure 7:**
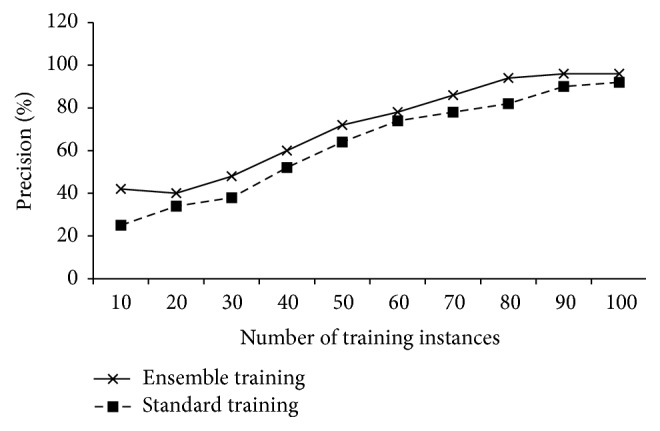
Precisions of ensemble training and standard training with increasing training instances.

We also evaluate the precisions of the ensemble training and the standard training with maximally 100 training instances. In Figure 7, it can be observed that the presented ensemble training strategy has better precision. And also, for the same precision, the ensemble training can reach to it earlier than that of the standard training.

**Figure 8:**
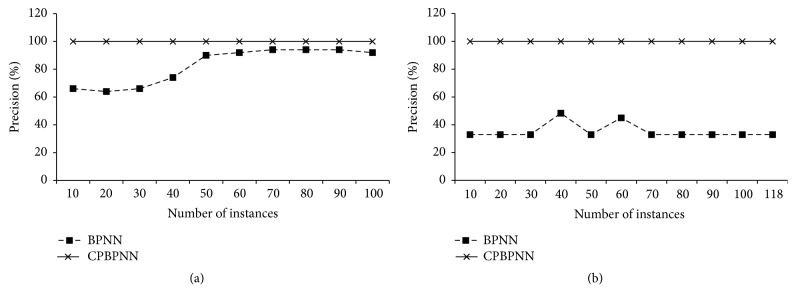
(a) CPBPNN precision for Iris dataset. (b) CPBPNN precision for Wine dataset.

The following evaluation is to observe the classification accuracy of CPBPNN with the ensemble and the cascading models. In this test we randomly selected 50 instances from the Iris dataset and 60 instances from the Wine dataset as the testing data. Therefore, the remaining 100 instances of the Iris and 118 instances of the Wine are used as the training data. We trained CPBPNN using an increasing number of the training instances. And also, in terms of comparison, a standard BPNN algorithm is implemented. Each instance is trained 200 times in both standard BPNN and CPBPNN. The result is shown in Figure 8.

Figure 8(a) shows the algorithm precision for the Iris dataset with the increasing number of training instances. It can be observed that for the standard BPNN algorithm, along with the number of the training instances increasing, the classification precision is increasing. Until the number is larger than 70, the precision is nearly stable. However, CPBPNN gives a remarkable result that even the number of training instances is small; it still gives 100% precision. By training only one class of data, the classifier has a strong ability to classify one instance belonging to the class. The instance that does not belong to the class cannot be recognized. Following the unclassified instance is cascaded into the second classifier until it is classified.  Figure 8(b) shows the precision for the classification using the Wine dataset. The figure indicates that the standard BPNN cannot deal with the dataset well. With only 200 times' training for each training instance, the standard BPNN gives not only low precisions but also unstable performances. The precisions are highly depending on the parameter values in the BPNN. For example, we trained each instance 4000 times instead of the 200 times used in the experiments; the precision reaches to 61.7%. However, CPBPNN still gives excellent performances. Even 10 training instances can result in 100% classification accuracy.

### 4.2. Efficiency

The efficiency evaluation focuses on the relationship between the algorithm running time and the volume of data. Therefore, we duplicate the Iris dataset from 1 MB to 1 GB to observe its processing time using the standard BPNN and the CPBPNN algorithms.

**Figure 9:**
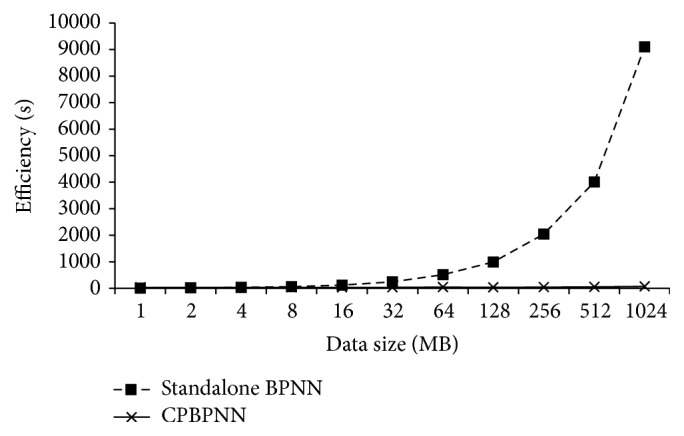
Efficiency comparison of CPBPNN and standalone BPNN.

Figure 9 shows that when the data size is small, both algorithms have low running time. Actually when the data size is less than 4 MB, the standalone BPNN outperforms CPBPNN due to the overhead of Hadoop framework. However, when the data size becomes large, the efficiency of the standalone BPNN deteriorates. Benefiting from distributed computing, CPBPNN performs with higher efficiency.

**Figure 10:**
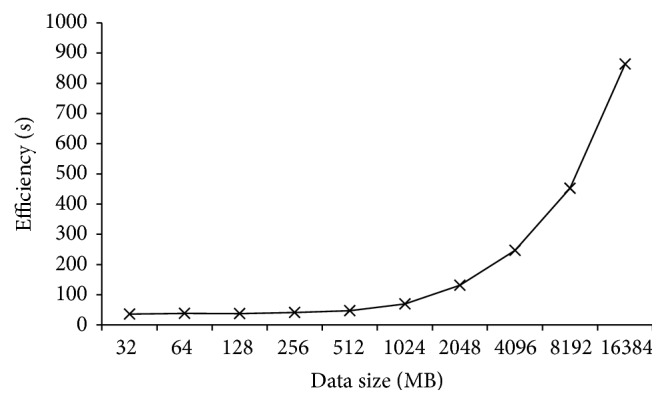
Efficiency of CPBPNN with increasing data size.

For further observing the efficiency of CPBPNN, we also duplicated the data size to 16384 MB.  Figure 10 shows that along with the data size increasing, the processing time of CPBPNN also becomes larger. But compared to the processing time of the standalone BPNN, CPBPNN gives better performances.

**Figure 11:**
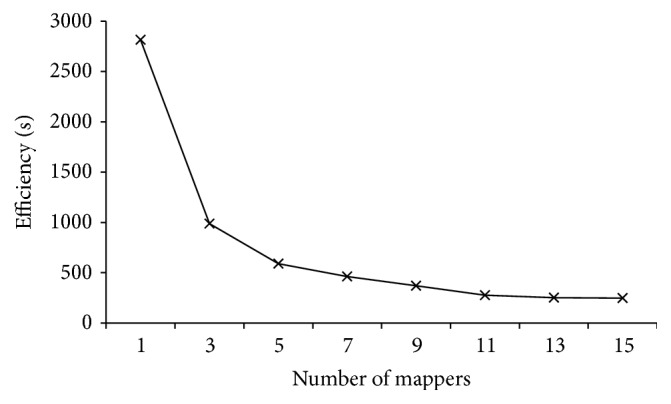
Efficiency of CPBPNN with increasing number of mappers.

Figure 11 shows the efficiency of CPBPNN with increasing number of mappers. The figure indicates that with more numbers of mappers, the algorithm performs better. However, when the number of mappers increases to a certain number, the efficiency enhancement becomes lower. The reason is that, in certain cases, two different numbers of mappers result in the same number of mapper processing waves, which cannot greatly improve algorithm efficiency. For example, 13 mappers need 5 waves, which is the same as the waves 15 mappers need. Therefore, although two more mappers are supplied, efficiency is not obviously improved.

### 4.3. Algorithm Comparison

For further study of the performance of CPBPNN, we also employ CPBPNN to process delta elevators dataset [33] and compare the result to the work [23, 24]. In our experiment, eight mappers are employed. In each network, 15 neurons are located in hidden layer. The number of the training instances is 4000 whilst the number of the testing instances is 5517. The result is shown in Table 3. In the table, *t*_training_ represents the training time; *t*_test_ represents the testing time; *S*_*t*_ represents the standard deviation of the error; *e*_RMSt_ represents the root mean square value of the error.

The table indicates that the classification precision of CPBPNN cannot maintain 100% accuracy. The accuracy is outperformed by those of FFH1b and ELM125. The reason is that, in the delta elevators dataset, certain classes contain less numbers of instances. Even a certain class only contains only one instance. Therefore the sub-BPNNs may not be sufficiently trained, which results in wrong classification. The table also shows that CPBPNN has lower efficiency because of the overhead of Hadoop framework. However, CPBPNN offers a way of dealing with the classification tasks with large volume of data.

## 5. Conclusion

This paper presents CPBPNN, a MapReduce based backpropagation neural network algorithm using cascading model. The algorithm mainly contributes to three phases, the speedup in the training phase, the speedup in the classification phase, and the precision improvement in the classification phase. In the training phase, the ensemble techniques including bootstrapping and majority voting have been employed. The ensemble based training strategy can train the network in high efficiency whilst maintaining satisfied accuracy. The classification is based on the data separation. By using a number of trained subneural networks, CPBPNN can process the classification with high efficiency when the data size is large. Each training step in the cascading model focuses on training only one class of the training data so that the classifier has a high accuracy on recognizing instances belonging to the class. Based on a number of cascading steps, CPBPNN can give an accurate classification result. The experiments evaluate CPBPNN in terms of precision, efficiency, and scalability. The experimental results indicate that the presented algorithm is suitable for dealing with classification tasks for large volume data. However, the algorithm also encounters one issue that Hadoop cannot perfectly support iterative operations. So the algorithm has to start and stop a series of mapper and reducer tasks, of which the overhead affects the algorithm efficiency. In the future, the algorithm implementation on Spark [34], an in-memory computation based distributed platform, should be studied. Its remarkable iteration-support mechanisms could supply further algorithm efficiency improvement.

## Figures and Tables

**Table 1:** The specification of the cluster.

Namenode	CPU: Core i7@3 GHz
	Memory: 8 GB
	SSD: 750 GB
	OS: Fedora

Datanodes	CPU: Core i7@3.8 GHz
	Memory: 32 GB
	SSD: 250 GB
	OS: Fedora.

Network bandwidth	1 Gbps

Hadoop version	2.3.0, 32 bit

**Table 2:** Data set details.

Type	Dataset characteristics	Instance number	Attribute number	Class number
Iris	Multivariate	150	4	3
Wine	Multivariate	178	13	3

**Table 3:** A comparison to FFH1b [23], ELM125, and SVR [24].

Algorithm	Running time (*t*_training_ + *t*_test_)	*S*_*t*_ (×10^−3^)	*e*_RMS*t*_ (×10^−3^)
FFH1b	2.50225	1.8	1.89
ELM125	0.38	1.54	1.54
SVR	1237.71	3	3
CPBPNN	332	1.95	1.95
